# Empathy and cultural humility: Caribbean medical students' experience in Taiwan's Silent Teacher family interviews

**DOI:** 10.1002/ase.70050

**Published:** 2025-05-27

**Authors:** Hsiang‐Chin Hsu, Tzu‐Ching Sung

**Affiliations:** ^1^ Department of Emergency Medicine, National Cheng Kung University Hospital, College of Medicine National Cheng Kung University Tainan Taiwan; ^2^ School of Medicine, College of Medicine National Cheng Kung University Tainan Taiwan; ^3^ School of Medicine for International Students, College of Medicine I‐Shou University Kaohsiung Taiwan

**Keywords:** anatomical education, cultural humility, empathy, ethical development, humanistic medical education, silent teacher

## Abstract

International medical students at I‐Shou University's School of Medicine for International Students (SMIS) receive Taiwan government‐funded scholarships to cultivate skilled and compassionate medical professionals from the Caribbean, Central America, and the Pacific Islands. This study examines the meaningful impact of Caribbean medical students' participation in interviews with the families of silent teachers, a central element of Taiwan's distinctive approach to anatomical education. Through these interviews, students were exposed to the deeply personal narratives of body donors, such as their life stories, motivations for donation, and their values, such as altruism, family devotion, and reverence for life. These interactions offered the students a rare opportunity to bridge the gap between technical medical training and healthcare's emotional, ethical, and cultural dimensions. This study examines reflective practices' impact on Caribbean medical students' development during interactions with Silent Teacher donors. Reflective narratives from 28 culturally diverse students were analyzed using thematic analysis. The experience enhanced the students' understanding of the significance of body donation in Taiwanese society, which contrasts with more anonymous approaches in Western medical education. As a result, international students commented on key professional attributes, including cultural humility, empathy, and a stronger ethical awareness. The family interviews allowed students to engage in the human aspect of medicine, reinforcing the importance of compassionate care and emotional intelligence in their future medical practice. This program is a meaningful model for integrating humanistic and ethical learning into the curriculum, especially for international students, fostering their growth into well‐rounded, culturally aware, and empathetic physicians.

## INTRODUCTION

Empathy, evolving through developmental stages—from passive recognition of others as living beings to spontaneous perspective‐taking, and culminating in ethical responsibility—enhances self‐awareness and emotional intelligence. It forms the foundation for personal development and fosters effective interpersonal relationships.^1^ Empathy supports intercultural competence by integrating principles, such as global respect, mutuality, and trust.^2^ As emphasized in the framework of cultural humility,^1^ this progression requires a lifelong commitment to self‐reflection and self‐critique, addressing power imbalances and forming non‐paternalistic partnerships. These qualities hold significant potential for higher education, particularly in leveraging healthcare education's focus on culturally competent care to inspire similar approaches across disciplines.^1^ As a cornerstone of both personal and professional growth, empathy not only deepens self‐awareness and emotional insight but also strengthens the ability to provide compassionate, patient‐centered care—an essential hallmark of excellence in clinical practice.^3^, ^4^

Anatomy education has traditionally served as a cornerstone of medical training, focusing on understanding the human body's structure. However, contemporary approaches increasingly emphasize integrating humanistic elements into the curriculum to enrich the learning experience. The personalization of body donors—acknowledging their humanity and spiritual dimensions—has been shown to foster empathy, ethical awareness, and professionalism among medical students.^5^ This approach contrasts with the conventional practice of anonymizing donors, which, while practical, often neglects the profound contributions of their lived experiences to the educational process.^5^, ^6^ By incorporating donors' narratives, anatomy education not only enhances students' comprehension of topographical anatomy but also aligns with the biopsychosocial model of healthcare. This model advocates a holistic understanding of individuals, integrating their physical, emotional, and social dimensions. Treating body donors as mentors or first patients allows educators to instill a deep respect for the altruistic act of donation, fostering a culture of compassion and ethical responsibility among future healthcare providers.^5^, ^6^ This shift underscores the evolving role of anatomy education, bridging technical expertise with the cultivation of empathy and professionalism essential for patient‐centered care.

The Silent Teacher Program represents a transformative shift in medical education by integrating humanistic values into the study of anatomical sciences.^7^ Traditional cadaveric study methods, while effective in fostering technical proficiency, often prioritize anatomical knowledge at the expense of personal connection to the human body. This technical focus can inadvertently contribute to the depersonalization of donors, fostering clinical detachment among medical students.^8^ Research suggests that detachment in medical training may contribute to a reduction in empathy as students' progress through their education, potentially hindering the development of professional identity formation, which is essential for cultivating compassionate and patient‐centered care. To mitigate this, contemporary anatomy education integrates educational theories, ethics, and humanistic perspectives, reframing anatomy as an early clinical experience. This approach fosters a transformative learning environment that enhances anatomical understanding while reinforcing empathy, compassion, and humanistic medical practice.^9^ Conceptualizing anatomy as a “relational practice,” where students engage not only with body donors but also with the ethical and historical dimensions of anatomical education, reinforces professional identity formation and humanistic learning.^10^ The relational anatomy model emphasizes respect for human beings both in life and death, recognizing the intrinsic dignity of all individuals. Addressing this issue requires a curriculum that integrates both objective scientific knowledge and subjective human experiences, as seen in initiatives that combine humanities and sciences to foster empathy.^11^

The Silent Teacher Program promotes an ethical and humanistic approach, encouraging students to view donors not merely as anatomical specimens, but as individuals who lived meaningful lives and made meaningful contributions to medical education through their selfless donation. This perspective allows students to develop both technical skills and moral awareness, enhancing emotional engagement during their training. The program bridges the gap between technical proficiency and humanistic care, fostering clinicians who are both emotionally engaged and morally aware,^12^ ensuring that technical skills are developed alongside compassion and empathy.^13^

The Silent Teacher Program traces its origins to 1995, when Ms. Lin, a dedicated donor at Mahāyāna Buddhist Tzu Chi Hospital in Hualien, made the first body donation specifically for medical education. This remarkable act of altruism laid the foundation for a program that is now internationally recognized for its emphasis on respect, dignity, and ethical responsibility in medical education. The program's structured seven‐step process ensures that donated bodies are treated with the utmost respect throughout their educational use. Key components of this process include honoring the donor through ceremonial events, facilitating interactions with the donor's family, and incorporating reflective exercises that prompt students to contemplate their ethical responsibilities toward the donated body.^7^

A distinctive feature of the Silent Teacher Program is its emphasis on the donor's life and familial connections, fostering meaningful relationships between medical students and the families of the donors. This focus contrasts sharply with the anonymity and clinical detachment that often characterizes Western medical education's body donation programs. By incorporating these humanistic elements, the program enriches students' anatomical learning experience while also promoting empathy and moral reflection. This holistic approach not only deepens medical students' understanding of anatomy but also instills a greater sense of ethical responsibility, ensuring that medical education is grounded in respect for both the human body and the life it once supported.^14^

This study explores the impact of the Silent Teacher Program at I‐Shou University on the personal and professional development of Caribbean medical students, with an emphasis on their professional identity formation. Personal development is conceptualized as the enhancement of self‐awareness, empathy, and ethical reflection, fostering the students' capacity to engage with diverse cultural and moral perspectives. Professional development involves integrating these attributes into their emerging roles as physicians, equipping them with the skills to provide culturally competent, empathetic, and ethically grounded care. By participating in interviews with the families of body donors—a distinctive aspect of Taiwan's anatomical education—students gained deeper insights into the values and motivations behind body donation. Through qualitative analysis of student reflections, the study examines how these experiences introduce empathy, cultural humility, and ethical awareness, highlighting the program's role in fostering both technical proficiency and humanistic qualities in future clinicians.

## METHOD

### Study design and participants

This study employed a qualitative design, utilizing narrative reflections provided by Caribbean medical students participating in the Silent Teacher Program. The core data sources consisted of three reflective documents written by students after visiting the families of individuals who donated their bodies to medical education. These documents offered rich, narrative accounts that captured the emotional, ethical, and professional dimensions of the students' experiences. The analysis of these reflections, conducted using thematic analysis, provided insight into the meaningful influence the program had on the students' emotional and professional development.

The participants consisted of 28 first‐year and second‐year Caribbean medical students, including 27 from Caribbean nations and one from Tuvalu, enrolled at the SMIS, I‐Shou University, Taiwan. These students, originating from diverse cultural and educational backgrounds, had completed their foundational years of medical training. The anatomy curriculum at the SMIS integrates the use of two Silent Teachers (body donors), with one donor dedicated per semester, to elevate the quality of students' anatomical education. These 28 students, representing 27 Caribbean nations and Tuvalu, as shown in Table 1, are participants in a specialized medical education initiative funded by scholarships under the Taiwanese government's Humanitarian Assistance program, rather than part of exchange programs. Collectively, they constitute 12.5% of the total classes (1 out of 8) within the School of Medicine and contribute 20% of the total teaching hours (1/5). Their active participation reflects a focused effort to deliver a rigorous and customized learning experience, addressing their unique educational needs and equipping them to fulfill their professional aspirations.

**TABLE 1:** Age statistics by year, nationality, and gender in the Silent Teacher Program participants.

Year	Nationality	Gender	*N*	Mean	SD	Min	Max	Median
1	Belize	Female	1	28.0	–	28	28	28
1	St. Lucia	Female	6	23.0	2.8	19	26	23
1	St. Lucia	Male	2	31.0	4.2	28	34	31
1	St. Vincent & the Grenadines	Female	1	25.0	–	25	25	25
1	Tuvalu	Male	1	31.0	–	31	31	31
2	Belize	Female	5	25.4	1.7	24	28	25
2	Belize	Male	3	28.0	3.0	25	31	28
2	St. Lucia	Female	8	27.4	4.7	21	35	28.5
2	St. Lucia	Male	1	32.0	–	32	32	32

As part of their medical humanities curriculum, students participated in three separate home visits with the families of individuals who had donated their bodies to the Silent Teacher Program. Each visit corresponded to a different Silent Teacher. During these sessions, family members had the option to share personal narratives about the donors, offering a unique balance between anonymization and personalization. This approach aligns with recommendations in anatomical education literature, which advocate for allowing donors to voluntarily provide personal information to foster empathy and understanding while maintaining respect for donor privacy.^15^

Conducted over several months in 2023 during the summer break, the visits provided students with a meaningful opportunity to connect with the altruistic motivations and cultural values underlying body donation. Through these interactions, students developed a deeper appreciation of the ethical and humanistic dimensions of medical education, enriching their perspectives and fostering a holistic approach to their future roles as compassionate and culturally sensitive healthcare providers. Participation in the Silent Teacher family visit program was voluntary, with a total of 28 students taking part. To accommodate scheduling constraints for both families and students, the visits were organized into small groups of approximately 8 to 12 students per session. Each student participated in at least one visit, ensuring full cohort representation. Coordination of the visits, including communication with the families and arrangement of meeting dates and times, was managed by domestic medical students. Depending on the academic calendar, these visits occurred either before or after students completed their anatomy coursework. Medical students typically interacted with Silent Teachers during the first semester, while post‐baccalaureate students engaged with them in the second semester. As a result, some students experienced family visits or cremation ceremonies involving different Silent Teachers than those used in their dissection courses.

Each visit was designed to facilitate meaningful and reflective dialogue, typically lasting 1–2 h. The sessions included in‐depth discussions about the donors' lives, the motivations behind their body donation, and the experiences of their families. The structured meetings often involved group discussions, family‐led presentations, and interactive sessions where students could ask questions. Since international and local students attended anatomy courses at different times but worked with the same body donors, local students also participated in the family interviews. In cases where international students encountered language barriers, local students provided translation support to facilitate clear communication. To maintain consistency in understanding among participants, the interviews were conducted in small groups, minimizing potential discrepancies in interpretation.

Following each visit, a group reflection was collaboratively produced by the participating students within 1 week of the experience. Students convened shortly after the visit to discuss their thoughts and emotions while the memories were still vivid. This timely process helped preserve the emotional immediacy and authenticity of their responses. In total, three group reflective narratives—one from each visit—were collected, representing the shared perspectives of all 28 students who took part in the program. These documents, written in English, were analyzed using thematic analysis to identify key themes, such as empathy, cultural humility, and ethical awareness. To address potential biases—such as those related to language translation, group dynamics, and the emotional intensity of the visits—each session was carefully facilitated and followed by a structured debriefing. These debriefing sessions provided students with a supportive space to process their experiences, fostering deeper emotional engagement and contributing to more nuanced and meaningful reflections. This experiential learning activity not only enriched students' understanding of the ethical principles and humanistic values integral to medical practice but also enhanced their ability to navigate the complex cultural and emotional dimensions of patient care.

### Context and setting

The Silent Teacher Program is a distinctive educational initiative in Taiwan where individuals donate their bodies posthumously for medical education. This program offers a more humanistic approach to anatomy education by fostering a relationship between medical students, the donors, and the families of the silent teachers. The goal was to instill values of empathy, respect, and ethical responsibility in future physicians.

The data for this study were collected following students' visits to the homes of the families of individuals who had donated their bodies to I‐Shou University's medical school. The visits took place in Gangshan District, Kaohsiung City, and Chiayi County, Taiwan, between July and August 2023. During these visits, students had the opportunity to engage in intimate conversations with the families, learning about the silent teachers' lives, their motivations for body donation, and the impact of these donations on the families.

Given that most meetings with family members are scheduled during the summer vacation, the timing of home visits is carefully coordinated to align with students' availability while accommodating the schedules of the families. These visits are not conducted simultaneously but are instead organized in batches to ensure flexibility and convenience for all participants. The process begins with assisting family members in confirming their preferences, including the date, time, and any specific details of the meeting. Once arrangements are finalized, local medical students from the School of Medicine, along with voluntary participants from the SMIS, attend these meetings at the designated locations, facilitating meaningful interactions between the students and the families.

The selection of Silent Teachers for these activities typically follows a one‐year cycle and is contingent on availability. As a result, the Silent Teachers involved in home visits may differ from those participating in cremation ceremonies. This variation arises due to the dynamic allocation of donors within the anatomical education curriculum, reflecting the program's structured yet flexible approach to donor utilization and student engagement.

### Data collection

Data were collected from the written reflections of the 28 students, collected anonymously in a group setting to minimize social desirability bias and promote candid responses. Open‐ended prompts were employed, offering participants the freedom to express a diverse range of emotions and perspectives. These reflections were guided by Data S1, which provided structured prompts asking students to articulate their emotional responses, ethical considerations, and the broader professional insights gained from visiting the Silent Teachers' families. The reflections covered three distinct visits.

A visit to the family of one Silent Teacher on August 19, 2023, where students learned about the donor's life story, from their upbringing in a small village to their role as a mother and community member, and their eventual decision to donate their body to medical education.

A visit to the family of another Silent Teacher on July 26, 2023, where students engaged with the family of a donor who had dedicated his life to his community and family, and whose generosity extended to his decision to donate his body after battling prostate cancer.

A visit to the family of a third Silent Teacher on July 16, 2023, where students reflected on the donor's sacrifices, his foresight in preparing his body for donation, and his family's pride in his altruistic actions.

The reflections provided rich, qualitative data that captured the students' personal growth, ethical insights, and professional reflections on the importance of empathy and humanism in medical practice.

### Data analysis

The reflective narratives were analyzed using thematic analysis, a rigorous qualitative method that allows researchers to systematically identify, organize, and interpret patterns within qualitative data.^16^ This analytic approach was particularly well‐suited for this study, as it enabled the identification of key themes related to the students' emotional and ethical growth, as well as their professional development through the Silent Teacher Program. The thematic analysis followed a six‐phase process with the aid of ATLAS.ti software:

1. Familiarization with the data

The first stage involved a close reading of all 28 student reflections to gain a deep understanding of the students' personal experiences, the emotional impact of the Silent Teacher visits, and their interpretations of the broader ethical and professional implications. This step ensured that the researchers were fully immersed in the data.

2 Generating initial codes

A thorough coding process was then applied. Segments of text that captured significant ideas related to emotional responses, ethical dilemmas, and professional values were highlighted and labeled. For instance, emotional reactions to the act of body donation, such as feelings of gratitude, reverence, and empathy, were consistently coded across multiple reflections.

3 Development of thematic framework

The final stage of the coding process involved synthesizing the sub‐themes into overarching thematic domains, forming a hierarchical structure that encapsulated the primary findings. The final thematic framework was aligned with the thematic patterns reported in Data S2.

4 Reviewing and refining themes

After the initial themes were identified, they were reviewed to ensure they accurately captured the depth and breadth of the students' reflections. The research team re‐read the reflections to refine the themes and ensure coherence across the data.

5 Defining and naming themes

The final themes were clearly defined and named to reflect the essence of the students' experiences. For example, the theme of Emotional Reflection was defined as the students' emotional journeys through the Silent Teacher Program, encompassing feelings of empathy, respect, and human connection to the deceased.

6 Contextualizing themes within literature

Finally, the themes were compared and contextualized within existing medical education literature. The analysis highlighted the Silent Teacher Program as a unique educational tool that fosters humanistic values, such as empathy, respect, and ethical reflection, which are crucial for developing compassionate and ethically responsible physicians.

### Rigor and trustworthiness

To ensure the rigor and trustworthiness of the findings, several strategies were employed:

1. Data triangulation

The reflections from different student cohorts and visits were compared to ensure the consistency of the emerging themes. This approach allowed for a deeper understanding of the program's impact across multiple perspectives.

2 Investigator triangulation

The two authors of this study, HCH and TCS, participated in the coding process. Independent coding was performed, and discrepancies in theme identification were resolved through discussion and consensus, thereby reducing researcher bias and enhancing the reliability of the results.

3 Reflexivity

Researchers maintained reflexive journals throughout the data analysis process to document their thoughts, biases, and assumptions. The research team consisted of individuals with diverse academic and cultural backgrounds, including expertise in medical education, qualitative research, and intercultural communication. This positionality provided both opportunities and challenges; while diversity offered valuable perspectives, it also necessitated a careful examination of potential biases that could arise from differing cultural or professional frameworks.

The theoretical lens adopted in this study was informed by constructivist and humanistic principles, emphasizing the subjective experiences of participants and the role of reflection in professional identity formation. Researchers engaged in ongoing self‐reflection, noting how their assumptions about medical education and cultural practices could shape their interpretation of the data.

Key insights documented in the reflexive journals included the recognition of potential biases when analyzing narratives from culturally diverse participants. For example, researchers noted the importance of avoiding overgeneralizations about cultural values and focusing instead on individual experiences. These reflections guided the coding and analysis process, ensuring that the findings were grounded in the data rather than influenced by preconceptions.

This reflexivity helped ensure that the analysis was grounded in the data rather than influenced by the researchers' preconceptions. By explicitly acknowledging and addressing these factors, the research team aimed to enhance the credibility and trustworthiness of the study's findings.

4 Peer debriefing

To further enhance the credibility of the findings, peer debriefing sessions were conducted. Three external experts in qualitative research and medical education were consulted to review the thematic analysis and offer feedback on the coherence and validity of the themes. Their input helped ensure that the analysis was robust and reflected the experiences of the students accurately.

### Ethical considerations

Ethics approval for the study was obtained from the Institutional Review Board of I‐Shou University (IRB No.: 2024026), ensuring that all research activities adhered to the ethical guidelines for studies involving human participants. All participating students provided written informed consent for their reflections to be used for research purposes. Students were explicitly informed that participation in the study was voluntary and that they could opt out of having their reflections included in the research without any impact on their educational experience or assessments.

The families of the silent teachers graciously consented to the educational visits and the subsequent use of their shared narratives for this study, with family members allowed to share personal stories at their discretion voluntarily. During the informed consent process, families were given the choice to have their names included in the study or to remain anonymous. For many, publicly recognizing their loved ones' contributions was regarded as an honor and a meaningful tribute to their altruistic donation. In these instances, names were included only with explicit consent. These narratives were thoughtfully integrated into the students' reflections to enhance their learning experience while adhering to strict ethical standards.^15^ To safeguard the privacy and dignity of the participants and the silent teachers' families, when families opted for anonymity, all identifiable information not voluntarily disclosed by the families was anonymized in the final report. Meticulous care was taken to ensure that the students' reflections were respected, especially considering the sensitive nature of body donation and the deep personal stories shared by the silent teachers' families.

## RESULTS

Table 1 shows the age distribution of first‐year and second‐year students, categorized by nationality and gender. Among the first‐year students, the single female participant from Belize was 28 years old, while St. Lucian females had a mean age of 23.0 years (SD = 2.8), with male participants from St. Lucia being notably older, having a mean age of 31.0 years (SD = 4.2). A female from St. Vincent & the Grenadines and a male from Tuvalu were aged 25 and 31, respectively. In the second‐year cohort, Belizean females had a mean age of 25.4 years (SD = 1.7), and the males had a mean of 28.0 years (SD = 3.0). The mean age of St. Lucian females increased to 27.4 years (SD = 4.7), while the only male participant from St. Lucia was 32 years old. The data reveal that male participants were generally older than their female counterparts across both first‐year and second‐year groups.

The findings are organized around three central themes that emerged from the analysis of the students' reflections on their experiences in the Silent Teacher Program. These themes, emotional reflection, ethical development, and professional identity formation, highlight the transformative impact of the program on students' understanding of the humanistic aspects of medicine. Through these themes, the reflections reveal deep insights into how future medical practitioners internalize values, such as empathy, respect for life, and ethical responsibility, all of which are essential components of professional growth in medical education. The results also include a quantification of the recurrence of these themes, based on the number of reflections that referenced each theme, providing a clearer picture of their prevalence across the cohort. Table 2 summarizes these themes and sub‐themes, using descriptive qualifiers to indicate their prominence in the reflections.

**TABLE 2:** Themes and sub‐themes identified in student reflections.

Theme	Sub‐theme	Illustrative presence
Emotional reflection	Gratitude and respect	Widely expressed
	Humility and awe	Commonly expressed
Ethical development	Altruism and selflessness	Strongly evident
	Ethical responsibility	Frequently noted
Professional identity formation	Empathy and compassion	Predominantly emphasized
	Humanistic values in medicine	Deeply reflected

### Theme 1: Emotional reflection

The theme of emotional reflection encapsulates the students' emotional responses to the Silent Teacher Program, particularly their reactions to the generosity of the body donors and their interactions with the donors' families. This theme emerged across all student reflections, highlighting its centrality to the students' experiences and underscoring its significance in shaping their perspectives.

Many students expressed meaningful gratitude for the silent teachers, emphasizing how these donations extended beyond simple acts of generosity to embody the ultimate gift of knowledge. The reflections frequently described the emotions elicited by learning about the donors' personal lives, their altruism, and the sacrifices they made. For example, one student wrote:

Meeting the family of Mr. Lin and hearing about his decision to donate his body moved me deeply. His desire to contribute to the education of future doctors, even after his passing, is something I will carry with me throughout my medical career.

These emotional reflections were not limited to gratitude; students also expressed feelings of humility and awe. As one student put it:

The visit to the Chen family helped me realize the significance of what we are learning. It's not just about anatomy; it's about understanding life and death in a deeply human way.

The emotional responses varied from student to student, but they consistently emphasized the connection between the physical body and the life stories behind it, reinforcing the importance of empathy and respect in medical practice.

#### Sub‐theme: Gratitude and respect

Gratitude was the most frequently cited sub‐theme, appearing in 24 out of 28 reflections. Students consistently expressed gratitude not only for the opportunity to learn from the silent teachers but also for the trust placed in them by the families of the donors.

Exemplar quote:

Mr. Lin's donation of his body was driven by his belief that even in death, his body could contribute to the education of future physicians, allowing them to learn from his condition.

This quote exemplifies the altruistic and forward‐thinking mindset reflected in the reflections, emphasizing the deep respect for life and the enduring commitment to advancing medical education through such selfless acts.

#### Sub‐theme: Humility and awe

The sub‐theme of Humility and Awe appeared in 20 out of 28 reflections. Students described how their interactions with the silent teachers' families provided a humbling reminder of the humanity behind the donors they were studying.

Exemplar quote:

Mr. Chen's decision to become a silent teacher, despite his personal health challenges, reflects profound humility and an awe‐inspiring dedication to the betterment of future generations.

This quote encapsulates the transformative impact of the silent teacher program, fostering a deep sense of humility and reverence among students for those who make such extraordinary contributions to medical education.

### Theme 2: Ethical development

The ethical development theme captures the students' reflections on the ethical implications of body donation and the responsibilities of medical professionals in respecting the dignity of both living patients and deceased donors. This theme was referenced throughout the reflections.

Students frequently grappled with the ethical dimensions of body donation, particularly the concept of altruism. The silent teachers were often described as role models for selflessness, with students reflecting on how the donations made them reconsider the ethics of medical education and practice. For example, one student reflected:

Knowing that someone chose to give their body for our education has made me rethink my responsibilities as a future doctor. It's not just about learning the material; it's about honoring the gift that has been given.

Many reflections also touched on the contrast between the humanistic approach of the Silent Teacher Program and the more traditional, anonymous cadaveric dissection in Western medical education. Several students commented on how the program challenged them to think about the ethical dimensions of dissection, not as a technical exercise, but as an act that requires sensitivity, compassion, and respect for life.

#### Sub‐theme: Altruism and selflessness

The sub‐theme of altruism and selflessness was prominent in 23 out of 28 reflections, with students noting how the silent teachers' actions exemplified the highest ethical standards in medicine. One student wrote:

The silent teachers teach us more than anatomy; they teach us about selflessness and what it means to give something of great value to others.

#### Sub‐theme: Ethical responsibility

Ethical responsibility was another key sub‐theme, appearing in 21 out of 28 reflections. Students highlighted how the program instilled a sense of moral duty, not only to their patients but also to the donors and their families. As one student observed:

As future doctors, we must carry the responsibility of treating both living and deceased individuals with respect. The Silent Teacher Program has reinforced that ethical duty in ways I never expected.

I now understand that my ethical duty as a physician extends beyond the living patients I treat; it also includes respecting the bodies of those who have contributed to my education.

This reflection highlighted students' growing awareness of their broader ethical responsibilities—not only to their patients but also to the donors and their families. Such reflections are essential for cultivating a professional identity grounded in ethical integrity and compassion.

### Theme 3: Professional identity formation

The third major theme, professional identity formation, was evident in 95% (27/28) of the reflections. This theme explores how the Silent Teacher Program contributed to shaping the students' professional identities by reinforcing the importance of empathy, compassion, and respect in medical practice.

Many students described the program as a pivotal moment in their medical education, one that fundamentally altered their approach to patient care. One reflection noted:

Before this program, I saw medicine as primarily technical, but now I understand that being a good doctor requires more than just skill; it requires empathy and respect for every life, living or deceased.

The program's emphasis on humanistic values resonated with students, leading many to reflect on how these values would guide their future practice. As one student summarized:

The Silent Teacher Program has shaped not only how I see anatomy but how I see myself as a future physician. I now understand that our role is not just to treat disease but to care for people with compassion and respect.

#### Sub‐theme: Empathy and compassion

The sub‐theme of empathy and compassion was mentioned in 25 out of 28 reflections. Students consistently described how the program deepened their understanding of empathy and how it would influence their approach to patient care. One student stated:

This experience has taught me that empathy is not just about understanding others' feelings; it's about recognizing the dignity of every person, whether they are alive or deceased.

#### Sub‐theme: Humanistic values in medicine

The sub‐theme of humanistic values in medicine appeared in 24 out of 28 reflections, highlighting students' recognition of compassion, respect, and ethical responsibility as integral to medical practice. One student reflected:

The Silent Teacher Program allows us to know the social relation and the lived life of the mentor and gives us the opportunity to build a relationship with the body donor and the family which can extend into the future.

Many students emphasized that the Silent Teacher Program deepened their understanding of medicine beyond technical skills, reinforcing the importance of empathy, ethical reflection, and honoring the contributions of body donors. Through these experiences, students gained a broader perspective on the moral and emotional dimensions of patient care, underscoring the need of humanistic approaches in clinical practice.

In synthesizing these findings, the Silent Teacher Program provides a transformative educational experience for medical students. By integrating emotional, ethical, and professional development, the program enables students to move beyond technical skill acquisition and embrace a holistic, humanistic approach to medical practice. The high prevalence of the themes across all student reflections suggests that the program has a meaningful and consistent impact on students' professional growth.

## DISCUSSION

The Silent Teacher Program is a pioneering approach in medical education, uniquely integrating emotional, ethical, and professional dimensions into the learning experiences of medical students. This study explored the meaningful impact of the program on students' professional identity formation by analyzing their reflective accounts of visits to the families of silent teachers. These experiences, wherein students engaged directly with the families of individuals who donated their bodies to medical education, not only enhanced their technical knowledge but also deepened their understanding of empathy, altruism, and ethical responsibility in medical practice. This study confirms the program's success in cultivating these qualities and suggests that the Silent Teacher Program provides a holistic and humanistic approach to medical education, countering the dehumanization often observed in traditional, technical‐focused curricula.^8^, ^12^, ^14^

### Emotional reflection: Gratitude, humility, and awe

A key finding from this study is the significant emotional reflection observed in students' narratives, primarily expressed through feelings of gratitude, humility, and awe. These emotional responses were consistently present across all student reflections, underscoring the program's capacity to evoke deep emotional engagement in the learning process. The recurring expression of gratitude toward body donors highlights the role of the “silent teachers” in humanizing anatomical dissection. This transformation allows students to perceive donors not merely as anonymous teaching tools but as individuals who made a meaningful, selfless contribution to their education.

Existing literature supports the critical role of emotional reflection in medical education for cultivating empathy and humanistic care.^17^ Hafferty (1991) discussed the risk of depersonalization in cadaver dissection, where medical students may begin to view donors as objects, potentially leading to emotional detachment.^18^ Programs, such as the Silent Teacher initiative, address this challenge by bridging the divide between technical and emotional learning. Fostering students' recognition of the human identity behind the body cultivates empathy, respect, and an intense connection to human experience throughout the learning process. As highlighted in Commemorations and Memorials: Exploring the Human Face of Anatomy, the integration of commemorative practices into anatomical education underscores the importance of acknowledging the humanity of body donors to enrich the educational journey.^19^, ^20^

Gratitude, as a specific emotion, has been deeply linked to increased empathy and prosocial behavior, qualities essential for medical professionals. Similar to the reflections seen during Yale's Anatomy Ceremony, where medical students express their meaningful gratitude toward the individuals who selflessly donated their bodies, research indicates that such expressions of gratitude lead students to engage more deeply in compassionate care, showing greater respect for the dignity of their patients.^21^, ^22^ The gratitude students feel often extends beyond the anatomical learning experience, incorporating the ethical and emotional insights derived from their interaction with the donor's life story and the sacrifices made by their families. This understanding, much like the reflections shared at Yale's ceremony, is further enriched by cultural contexts, such as in Taiwan, where body donation carries significant moral and ethical weight.^19^

Moreover, students often reflect on humility and awe, which signify a shift in their perception of medical education and their evolving professional identity. The emotion of awe, often evoked by recognizing the magnitude of the donors' contributions, has been associated with enhanced moral and ethical reasoning in professionals.^23^ Through this experience, students express humility in acknowledging their limited understanding of life and death, learning to approach both with respect and reverence as part of their ongoing professional development.^24^

These emotional responses are consistent with findings from other studies that emphasize the value of incorporating emotionally charged experiences into medical education to foster self‐awareness, empathy, and ethical sensitivity.^19^ By confronting the reality of body donation in such a personal and emotionally intense context, students developed a deeper connection to their future roles as physicians, realizing the importance of treating patients holistically, rather than merely addressing their diseases.

### Ethical development: Altruism and ethical responsibility

The second major theme that emerged from this study was the ethical development of students, particularly their reflections on altruism and their ethical responsibilities as future physicians. The donations of the silent teachers were consistently framed as acts of meaningful altruism, challenging students to consider how they, as future doctors, could honor these sacrifices through ethically sound and compassionate medical practices.

This finding is consistent with the broader literature on ethical development in medical education, which emphasizes the importance of experiential learning in shaping ethical behavior and professional development.^25^ Altruism, often regarded as an abstract concept within medical ethics curricula, became a tangible reality for the students through their engagement with the families of silent teachers.^26^ Medical students who interact with the personal stories of body donors are more likely to internalize ethical principles, such as respect for autonomy and beneficence, as they confront the real‐life implications of these abstract ethical theories.^27^

The students' reflections on ethical responsibility were particularly significant, as many grappled with the moral obligation to treat the bodies of deceased individuals with the same respect and dignity afforded to living patients. This recognition of ethical duty is pivotal in shaping future medical professionals who are committed to upholding ethical standards in both clinical and educational contexts.^28^ Research suggests that engaging medical students in ethical reflection throughout their education fosters a strong sense of moral accountability, which is crucial for navigating the complex ethical challenges they will face in their professional lives.^29^

Furthermore, the program's emphasis on the ethical implications^26^ of body donation contrasts sharply with traditional anatomical dissection practices, where donors are often viewed as anonymous. The ethical dimension of body donation is frequently underplayed in these traditional settings. By involving the families of the silent teachers and sharing the donors' life stories, the program reinforces the humanistic and ethical aspects of medical practice.^14^ One student reflected:

I now understand that my ethical duty as a physician extends beyond the living patients I treat; it also includes respecting the bodies of those who have contributed to my education.

This reflection highlights the students' growing awareness of their broader ethical responsibilities—not only to their patients but also to the donors and their families. Such reflections are essential for cultivating a professional identity grounded in ethical integrity and compassion. The development of a moral and ethical framework is not just an academic exercise; it directly impacts the quality of care that future physicians will provide, contributing to a more empathetic and ethically sound medical practice.^29^

### Professional identity formation: Empathy, compassion, and humanism

The Silent Teacher Program has emerged as a transformative experience in shaping students' professional identity formation. It effectively integrates themes of empathy, compassion, and humanistic values, as seen in students' reflections. These reflections suggest that the program meaningfully influences their perspectives on their future roles as physicians, offering a unique counterbalance to the often technical and depersonalized aspects of medical education.

Empathy, widely regarded as a cornerstone of effective medical practice, is critical for patient‐centered care. However, numerous studies have documented a decline in empathy as medical students progress through their training. A comprehensive review demonstrates that empathy tends to erode due to the intense focus on technical skills and the depersonalization of patients in traditional medical education.^30^ This “empathy erosion” poses a challenge for fostering compassionate care. The Silent Teacher Program, however, counters this trend by providing students with emotionally rich and ethically charged experiences. The students' engagement with the families of body donors humanizes the learning process and reinforces the importance of empathy in medical care.^12^

Compassion is another crucial component in medical education that is often overlooked. Defined as understanding a patient's suffering and taking actionable steps to alleviate it, compassion has been shown to enhance patient outcomes, elevate patient satisfaction, and improve physicians' professional fulfillment.^31^, ^32^ The Silent Teacher Program excels in fostering compassion by highlighting the life stories of donors and nurturing emotional connections between students and donor families. These experiences help students internalize compassion as a core element of their professional identity, linking it with clinical excellence.^27^

Humanistic values, which are increasingly marginalized in modern medical education, are another critical aspect of the Silent Teacher Program. Studies have expressed concerns about the growing dehumanization within medical training, where the emphasis on disease management often eclipses patient‐centered care.^33^ The technical demands of medical education can lead students to focus predominantly on scientific knowledge and skills, often neglecting the human aspects of patient care. The Silent Teacher Program bridges this gap by reintroducing humanistic elements into the medical curriculum.^12^, ^14^ It reminds students that medicine is not only a science but also an art that requires empathy, compassion, and respect for the dignity of each patient.^23^

By offering a model for integrating humanistic education into medical training, the Silent Teacher Program aligns with contemporary goals in medical education, which emphasize the development of emotionally intelligent, ethically conscious physicians who are capable of delivering compassionate, patient‐centered care.^12^ This balance between technical proficiency and humanism is critical for producing well‐rounded physicians who are equipped to navigate both the scientific and emotional complexities of patient care.

### Implications for medical education

The findings from this study have significant implications for the future of medical education. The Silent Teacher Program serves as a powerful example of how medical schools can incorporate humanistic education into their curricula, ensuring that students develop not only the technical skills needed for medical practice but also the emotional intelligence, empathy, and ethical sensibilities required for compassionate care.

First, the program highlights the importance of integrating empathy and compassion into medical education. By allowing students to engage with the families of body donors and understand the personal stories behind the donations, the program fosters a deeper sense of empathy, which is essential for effective patient care. Medical schools should consider incorporating similar programs into their curricula to ensure that students develop the empathy needed to connect with patients on a human level, rather than merely focusing on disease treatment.

Second, the Silent Teacher Program emphasizes the need for ethical education that goes beyond theoretical discussions of ethics. By providing students with real‐life ethical dilemmas—such as the responsibility to honor the dignity of body donors, the program helps students internalize ethical principles and apply them in their professional practice. Medical schools should incorporate experiential learning opportunities, like those provided by the Silent Teacher Program, to ensure that students develop the ethical reasoning skills required for navigating the complexities of modern medical practice.

Finally, the program's focus on professional identity formation suggests that medical education should prioritize the development of humanistic values as a core component of professional growth. The Silent Teacher Program provides students with a unique opportunity to reflect on their role as future physicians and to develop a professional identity grounded in empathy, compassion, and respect for human dignity. Medical schools should consider implementing programs that facilitate this kind of reflection and identity formation, ensuring that students graduate not only as competent physicians but also as compassionate, ethically responsible professionals.

### Absence of negative themes

The absence of negative themes or reports of emotional distress in the students' reflections warrants careful consideration. While it is noteworthy that over 70% of the reflections identified positive themes, the lack of any negative concerns raises the possibility of response bias. Cultural norms or expectations may have influenced students to emphasize positive aspects, especially given the deeply respectful nature of the Silent Teacher Program in Taiwanese culture. Moreover, the voluntary nature of student participation may have predisposed participants to hold a favorable attitude toward the experience from the outset, thereby reducing the likelihood of negative feedback.

To further investigate this phenomenon, the methodology was critically examined to determine whether aspects of the data collection process may have constrained the expression of negative emotions or concerns. Reflections were collected anonymously and in a group format, which likely minimized social desirability bias and encouraged honest reporting. Additionally, open‐ended prompts were employed to allow participants the freedom to express a full range of emotions and perspectives. However, it remains possible that inherent cultural or contextual factors influenced the predominance of positive reflections.

Future iterations of the study could incorporate additional measures to ensure a comprehensive capture of student experiences. For example, conducting one‐on‐one interviews or employing qualitative methods that allow for a deeper exploration of emotional responses may uncover subtler or more nuanced aspects of the students' experiences. These modifications would help to balance the understanding of both the positive and potentially challenging dimensions of such deeply humanistic interactions, thereby enriching the evaluation of the Silent Teacher Program's impact on medical education.

### Limitations

This study has several limitations that must be acknowledged when interpreting the results. The relatively small sample size of 28 students from a single medical school in Taiwan limits the generalizability of the findings to other institutions, cultures, and medical education systems. Additionally, the study relied on self‐reported reflections, which may be subject to social desirability bias, as students could have been inclined to emphasize emotions, such as empathy, gratitude, and ethical awareness, in ways they believed would be favorably viewed by their instructors. Another limitation is the study's focus on the immediate impact of the Silent Teacher Program; the longer term effects on students' empathy, ethical reasoning, and professional identity formation remain unclear, and follow‐up studies would be needed to evaluate the program's lasting influence. Moreover, cultural factors were not explicitly explored in this study, despite the Caribbean background of the students. These cultural differences could have significantly shaped the students' perceptions and responses to body donation and ethical considerations. Furthermore, while some students participated in the family visits prior to their anatomy coursework and others afterward, our analysis did not stratify the data based on this timing difference. Thematic analysis was conducted on group‐level reflective narratives, each representing a mix of students with varying levels of prior anatomy experience. Although the timing of exposure to anatomy may have shaped students' interpretations and depth of reflection, a comparative analysis was beyond the scope of this study. In light of this limitation, future studies should consider systematically examining how the sequence of anatomy instruction and reflective practice influences students' ethical engagement and emotional responses.

More broadly, future research should address these limitations by including larger, more diverse samples, employing mixed methods, such as interviews or observations to triangulate data, and conducting longitudinal studies to assess the program's sustained effects on professional growth and ethical practice in different cultural and educational contexts.

## CONCLUSION

The Silent Teacher Program offers a powerful educational experience that fosters the emotional, ethical, and professional development of medical students. By engaging directly with the families of body donors, students gain a deeper appreciation for altruism, empathy, and ethical responsibilities that are fundamental to compassionate medical practice. The program's emphasis on humanistic values, such as empathy, respect, and ethical reflection, provides a valuable model for medical schools seeking to integrate these qualities into their curricula. Further research is needed to explore the long‐term impact of the Silent Teacher Program and its applicability across different cultural and educational settings. Nevertheless, this study highlights the potential for innovative programs like the Silent Teacher Program to shape the next generation of doctors into not only skilled practitioners but also compassionate, ethically grounded professionals.

## AUTHOR CONTRIBUTIONS

**Hsiang‐Chin Hsu:** Writing – original draft; methodology; software; writing – review and editing. **Tzu‐Ching Sung:** Conceptualization; methodology; software; writing – review and editing; writing – original draft; supervision.

## CONFLICT OF INTEREST STATEMENT

The authors declare that they have no known competing financial interests or personal relationships that could have appeared to influence the work reported in this article. This article represents the author's personal views only.

## Supporting information


Data S1.
