# Supplementary material for: Empathy and cultural humility: Caribbean medical students' experience in Taiwan's Silent Teacher family interviews
Source: Anat Sci Educ. 2025 May 27;18(7):629–41. doi: 10.1002/ase.70050 (PMC12222578; doi:10.1002/ase.70050)
Supplement: Supplementary file 1 — Data S1. [file ASE-18-629-s001.docx]

**Supplementary data 1.** Structured reflection guide for medical students

**Phase 1: Foundational understanding**

This stage establishes students' knowledge of the Silent Teacher Program and its role in medical education.

**Guiding prompts:**

1. **Understanding the background**

- What are the objectives of the Silent Teacher Program in medical education?
- What were your initial impressions of body donation before this experience?

1. **Cultural perspectives**

- How is body donation perceived in your culture compared to Taiwan’s Silent Teacher Program?
- How has this experience influenced your views on body donation?

**Phase 2: Reflection**

Encourages exploration of emotions, ethical considerations, and professional identity development.

1. **Emotional reflection, gratitude and respect, and humility and awe**

- How did meeting the Silent Teacher’s family impact you?
- How did this experience shape your understanding of altruism and sacrifice?
- What aspect of the Silent Teacher’s story resonated with you most?
- Why is gratitude essential in this learning experience? How did you express it?
- How did this experience deepen your appreciation for body donors and their families?

1. **Ethical development, altruism and selflessness, and ethical responsibility**

- What responsibilities do medical students have in honoring body donors?
- How does the Silent Teacher Program compare to traditional cadaveric dissection?
- What ethical dilemmas arose, and how did you resolve them?
- How does the Silent Teacher’s decision embody selflessness in medicine?
- How did this experience reinforce medical students' ethical duties?

1. **Professional identity formation, empathy and compassion, and humanistic values in medicine**

- How did this experience shape your understanding of the physician's role?
- How did it reinforce empathy, respect, and compassion in patient care?
- What aspects of this experience will you carry forward in your medical career?
- What does empathy mean to you in the context of this experience?
- How does the Silent Teacher Program exemplify humanistic values in medical education?

**Phase 3: Final reflection and application**

Encourages students to synthesize key takeaways from the experience.

**Final reflection prompts:**

1. How has this experience influenced your growth as a medical student and future physician?
2. What advice would you give future students participating in this program?
3. How should this type of experience be integrated into medical education?
4. What is the most valuable lesson you learned from this experience?

**Supplementary data 2**. Hierarchical structure of thematic categories

| **Main theme** | **Sub-theme** | **Description** |
| --- | --- | --- |
| **Emotional reflection** | Gratitude and respect | Expressions of appreciation for others, acknowledgment of selflessness, and emotional connections fostered through interactions. |
|  | Humility and awe | Reflections on personal growth, recognizing the impact of experiences, and feeling humbled by others' stories and sacrifices. |
| **Ethical development** | Altruism and selflessness | Recognition of selfless acts, putting others first, and an appreciation for body donation and other humanitarian contributions. |
|  | Ethical responsibility | Engagement with ethical dilemmas, decision-making, and moral obligations within healthcare and social contexts. |
| **Professional identity formation** | Empathy and compassion | Understanding the well-being of others, building relationships, and reflecting on personal and professional values. |
|  | Humanistic values in medicine | Recognition of the importance of cultural, ethical, and spiritual influences in medical practice and patient interactions. |
